# The Temporary Ligature

**Published:** 1890-02-22

**Authors:** 


### THE TEMPORARY LIGATURE.

Dr. Thomas H. Manley, of New York, contributes an article to the *International Journal of Surgery* "on some of the uses of the temporary transfixion ligature." It is designated temporary, because it is not permitted to remain longer than some twenty-four hours or less, and the apparatus necessary is simply a needle and thread. But in taking up deep vessels, there must be a reliable needle holder to do work where the fingers are wanting in length and strength. Antiseptic silk is the best material for the ligature, and the needle must be well tempered steel, spear-pointed, and bowed according to the special region to be explored, the deep vessels requiring a long, obtusely-bent needle, and the superficial a short one with a rather sharp angle. Having selected the instrument, it is passed in, on as nearly a vertical line as possible, and sent down perpendicularly until it is reasonably certain that the base of the vessel is passed, when the heel of the needle is quickly made to describe the segment of a circle at as sharp an angle as possible, in order that the needle's point may participate in the movement of the hand and reach the surface again embracing as little of the tissues as possible. The *venæ comites* must always be included with the artery. When Dr. Manley first employed this ligature he secured it with a sliding knot, and released it when necessary by drawing on the free ends; but with experience he found these projecting ends in the way, and that division with the scissors answers the same purpose. Among cases in which this ligature may be used with advantage, there is mentioned amputation at the hip joint. After commencing the oval flaps and dissecting them back for an inch or two, the arterial pulsation could be plainly seen. The needle was inserted close to Poupart's ligament and the point carried down close to the bone. The needle after its emergence was cut free from the ligature, and everything within slowly and firmly compressed till the femoral pulse ceased. A reef-knot was then tied, the free ends of the ligature snipped off and the amputation proceeded with. On division, the femoral artery was found empty, and the operator was not encumbered by torniquet or elastic bandage, while the operation was almost bloodless. After the limb was disjointed and severed, the open mouth of the artery was ligatured as usual, and, before closing the flaps, the temporary ligature was cut, "and the blood above let on." The progress of the case and recovery were uneventful. A second case reported was an epithelioma of the lower lip. Usually the operator depends on an assistant for the control of hæmorrhage, by pressing the edges of the lip between the finger and thumb. In the case in question 25 drops of a four p. c. solution of cocaine were first injected. Then a needle with double ligature was passed through the lower lip close to the angle on each side, and firmly tied. The diseased parts were leisurely cut away, and not a drachm of blood lost. A third case was a police officer who suffered division of the tendons and arteries of the wrist. As the man was dying from hæmorrhage there was no time to try and find the retracted radial and ulna arteries. Instead of compressing the brachial, Dr. Manley applied the temporary ligature to the arteries, and an hour later found and ligatured the proximal and distal ends." In this instance the temporary "ligature gave us a breathing spell, an opportunity to apply antiseptic dressings, and to secure skilled assistance." Several other cases of the kind are reported. The advantages claimed for the temporary ligature are, the simple material, only a needle and thread required; it is not so painful as the Esmarch bandage; with the transfixion ligature on the principal trunk or its branches collateral circulation becomes quickly established, and hence the limb's vitality is spared. But there are objections. Venous trunks may be lacerated by the needle's point: nerves may be injured: the artery itself may be pierced. It is certainly a measure which cannot be attempted, without a good and practical knowledge of anatomy.

